# Hypoxic postconditioning promotes mitophagy against transient global cerebral ischemia via PINK1/Parkin-induced mitochondrial ubiquitination in adult rats

**DOI:** 10.1038/s41419-021-03900-8

**Published:** 2021-06-18

**Authors:** Haixia Wen, Luxi Li, Lixuan Zhan, Yunyan Zuo, Kongping Li, Meiqian Qiu, Heying Li, Weiwen Sun, En Xu

**Affiliations:** 1 Institute of Neuroscience and Department of Neurology of the Second Affiliated Hospital of Guangzhou Medical University and Key Laboratory of Neurogenetics and Channelopathies of Guangdong Province and the Ministry of Education of China, Guangzhou, 510260 P. R. China; 2 Department of Neurology, Guangzhou First People’s Hospital, School of Medicine, South China University of Technology, Guangzhou, Guangdong, China; 3 Department of Neurology, Affiliated Brain Hospital of Guangzhou Medical University, Guangzhou Huiai Hospital, Guangzhou, China; 4 Guangzhou Institutes of Biomedicine and Health, Chinese Academy of Sciences, Guangzhou, 510530 China

**Keywords:** Cell death in the nervous system, Stroke

## Abstract

Mitophagy alleviates neuronal damage after cerebral ischemia by selectively removing dysfunctional mitochondria. Phosphatase and tensin homolog (PTEN) induced putative kinase 1 (PINK1)/Parkin-mediated mitophagy is the most well-known type of mitophagy. However, little is known about the role of PINK1/Parkin-mediated mitophagy in ischemic tolerance induced by hypoxic postconditioning (HPC) with 8% O_2_ against transient global cerebral ischemia (tGCI). Hence, we aimed to test the hypothesis that HPC-mediated PINK1/Parkin-induced mitochondrial ubiquitination and promotes mitophagy, thus exerting neuroprotection in the hippocampal CA1 subregion against tGCI. We found that mitochondrial clearance was disturbed at the late phase of reperfusion after tGCI, which was reversed by HPC, as evidenced by the reduction of the translocase of outer mitochondrial membrane 20 homologs (TOMM20), translocase of inner mitochondrial membrane 23 (TIMM23) and heat shock protein 60 (HSP60) in CA1 after HPC. In addition, HPC further increased the ratio of LC3II/I in mitochondrial fraction and promoted the formation of mitophagosomes in CA1 neurons after tGCI. The administration of lysosome inhibitor chloroquine (CQ) intraperitoneally or mitophagy inhibitor (Mdivi-1) intracerebroventricularly abrogated HPC-induced mitochondrial turnover and neuroprotection in CA1 after tGCI. We also found that HPC activated PINK1/Parkin pathway after tGCI, as shown by the augment of mitochondrial PINK1 and Parkin and the promotion of mitochondrial ubiquitination in CA1. In addition, PINK1 or Parkin knockdown with small-interfering RNA (siRNA) suppressed the activation of PINK1/Parkin pathway and hampered mitochondrial clearance and attenuated neuroprotection induced by HPC, whereas PINK1 overexpression promoted PINK1/Parkin-mediated mitophagy and ameliorated neuronal damage in CA1 after tGCI. Taken together, the new finding in this study is that HPC-induced neuroprotection against tGCI through promoting mitophagy mediated by PINK1/Parkin-dependent pathway.

## Introduction

Transient global cerebral ischemia (tGCI), which is common in clinical conditions such as drowning, cardiac arrest, and cardiopulmonary bypass surgery, leads to selective neuronal death in the hippocampal CA1 layer [1]. Previously, we had reported that hypoxic postconditioning (HPC) with 8% O_2_ for 60–120 min could offer neuroprotection in CA1 when applied 1–2 days after 10 min of tGCI. The maximum protective effect of HPC is achieved with 120 min of hypoxia and 1-day interval between hypoxia and tGCI [2]. However, the underlying molecular mechanisms of HPC-mediated cerebral ischemic tolerance have not yet been completely elucidated.

Perpetuated mitochondrial impairment is a common consequence of cerebral ischemic insult progression, leading to the opening of mitochondrial membrane transition pore, the elevation of oxidative stress, and the induction of apoptosis [3]. Mitophagy, well-studied selective autophagy, plays an essential role in protecting neurons against cerebral ischemia/reperfusion injury by selectively removing impaired or dysfunctional mitochondria [4, 5]. Our previous studies revealed that tGCI caused mitochondrial injury in CA1 via activation of autophagy [6, 7]. Autophagy disruption led to insufficient degradation of autophagy substrates and exacerbated neuronal death in CA1 after tGCI [6, 7]. In contrast, HPC-promoted autophagosomes maturation and restored autophagic flux, thereby protecting CA1 neurons from tGCI [6]. Importantly, mitophagy-related mitochondrial clearance in the reperfusion phase was beneficial to ischemia/reperfusion-induced neuronal injury in C57BL/6 mice after transient middle cerebral artery occlusion (MCAO) and in cultured cortical neurons with oxygen-glucose deprivation-reperfusion [8]. Consistently, Mdivi-1, a mitophagy inhibitor, abolished the reduction of mitochondrial markers translocase of outer mitochondrial membrane 20 homolog (TOMM20) and cytochrome c oxidase subunit 4 isoform 1, increased infarct volumes and enhanced neurological deficit after transient MCAO in C57BL/6 mice [9]. However, excessive mitophagy may be detrimental for neurons [10]. It might trigger autophagic cell death and contribute to delayed neuronal damage following ischemic stroke [11]. Although the neuroprotective potential of mitophagy has been emphasized and the insufficient turnover of damaged mitochondria after tGCI has been reported, it remains unknown whether mitophagy is involved in HPC-induced neuroprotection against tGCI and how mitophagy is activated to alleviate CA1 neuronal death by HPC after tGCI.

So far, three main pathways have been identified in the regulation of mitophagy in mammalian cells: phosphatase and tensin homolog (PTEN) induced putative kinase1 (PINK1)/Parkin or Parkin-dependent pathway, and two Parkin-independent pathways, including NIX/B-cell lymphoma-2 (Bcl-2)/E1B-19KD-interacting protein 3 (BNIP3) and FUN14 domain containing 1 (FUNDC1) pathway [12]. Of these pathways, PINK1/Parkin-dependent mitophagy was of special concern in the progress of multiple neurological diseases, especially cerebral ischemia [9]. PINK1 is a mitochondrial serine/threonine kinase encoded by *PINK1/PARK6*. Under steady-state conditions, PINK1 is constitutively imported into mitochondria and rapidly cleaved and degraded in healthy mitochondria. In response to mitochondrial depolarization, PINK1 is stabilized and accumulated on the outer mitochondrial membrane (OMM). Moreover, PINK1 promotes the translocation of Parkin from the cytoplasm to damaged mitochondria and activates E3-ubiquitin ligase activity of Parkin [13–15]. Active Parkin could cause a myriad of substrates ubiquitination in the OMM [16] and subsequently lead to the recruitment of ubiquitin adaptor proteins and mitochondrial ubiquitination, thereby causing engulfment of depolarized mitochondria by autophagosomes [17]. Notably, primary hippocampal neurons undergo PINK1/Parkin-mediated mitophagy in response to mitochondrial damage [18]. In addition, a large body of evidence has demonstrated the critical role of PINK1/Parkin-dependent mitophagy under in vivo pathological conditions such as cerebral ischemia [19–21]. Wang et al. demonstrated that the activation of PINK1/Parkin pathway effectively ameliorates neuronal damage in the cortex and hippocampal CA1 region via mediating damaged mitochondrial clearance after MCAO [21]. Furthermore, PINK1/Parkin pathway plays a vital role in the interplay between hypoxia-related mitophagy and cerebral ischemia [19, 22]. Nevertheless, little is understood whether HPC promotes mitochondrial ubiquitination via activating PINK1/Parkin pathway, thus inducing mitophagy to attenuate neuronal injury after tGCI.

Herein we aim to investigate whether HPC induces cerebral ischemic tolerance by activating mitophagy. Further, we will illustrate that HPC promotes mitochondrial ubiquitination through activating PINK1/Parkin pathway to allow selective mitochondrial clearance, thereby offering neuroprotection in CA1 against tGCI.

## Results

### HPC activates mitophagy and alleviates neuronal death in hippocampal CA1 after tGCI

**Fig. 1:**
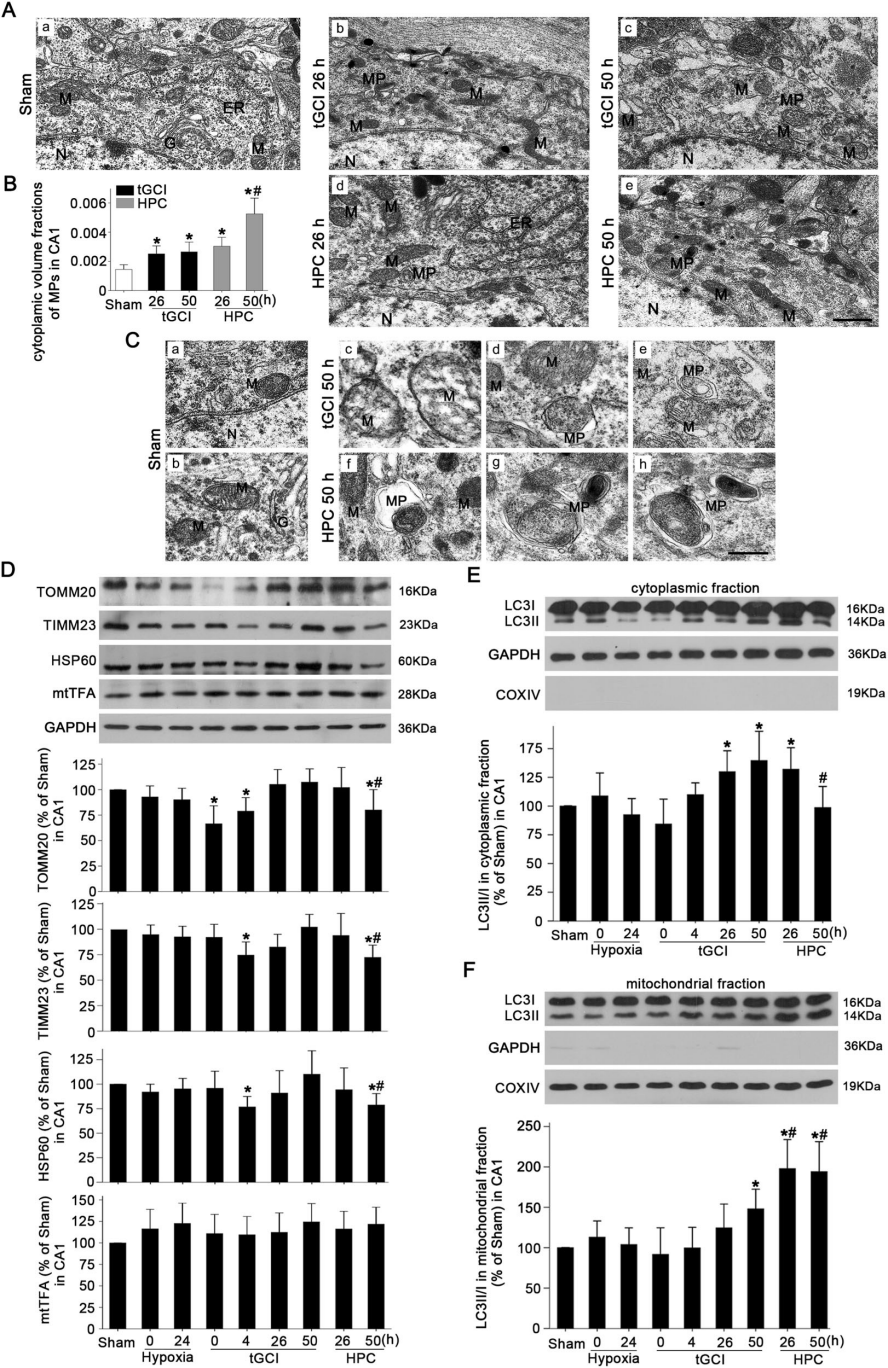
Effect of HPC on mitochondrial clearance in CA1 after tGCI. **A** TEM micrographs of CA1 neurons from Sham (a), tGCI (b) and HPC (d) groups at 26 h after reperfusion, tGCI (c) and HPC (e) groups at 50 h after reperfusion. Scale bar: 0.5 μm. N: nucleus; M: mitochondria; ER: endoplasmic reticulum; G: Golgi apparatus; MP: mitophagosome. **B** The cytoplasmic volume fractions of MPs in CA1 neurons from tGCI and HPC rats. Data expressed as percentage ± SD show the cytoplasmic volume fractions of MPs. **C** TEM micrographs of mitochondria ultrastructure in CA1 neurons from Sham rats (a&b) and MP ultrastructure from tGCI (c–e) and HPC (f–h) rats at 50 h after reperfusion. Scale bar: 0.25 μm. **D** Western blot analysis of TOMM20, TIMM23, HSP60, mtTFA in CA1. The histogram presents the quantitative analyses of TOMM20, TIMM23, HSP60 and mtTFA levels (*n* ≥ 4 in each group). Western blot analysis of LC3 in the cytoplasmic fraction (**E**) and mitochondrial fraction (**F**) of CA1. The histogram presents the quantitative analyses of LC3-II/I ratio. Data are expressed as percentage of the value of sham-operated animals. Each bar represents the mean ± SD. *p < 0.05 vs. Sham animals and ^#^*p* < 0.05 vs. tGCI group at the same time point.

First, mitophagy was examined by transmission electron microscopy (TEM). Micrographs of CA1 neurons from TEM in sham-operated (Sham) group displayed normal mitochondria with intact double membrane and dense and organized cristae (Fig. 1A, a; C, a, b), whereas neurons in tGCI groups showed a remarkable increase in swollen mitochondria with disorganized inner-membrane cristae and a few mitophagosomes at 26–50 h of reperfusion after tGCI (Fig. 1A, b, c; C, c–e). In contrast to tGCI groups, more mitophagosomes were observed in HPC groups at 50 h of reperfusion (Fig. 1A, e; C, f–h). Relative to Sham rats, the cytoplasmic volume fractions of mitophagosomes increased after tGCI with or without HPC, which further increased in HPC group at 50 h of reperfusion (Fig. 1B). Then, the levels of mitochondria-related proteins including TOMM20, translocase of inner mitochondrial membrane 23 (TIMM23), heat shock protein 60 (HSP60) and mitochondrial transcription factor A (mtTFA) in CA1 were measured. Compared with Sham group, TOMM20 level decreased at 0–4 h after reperfusion of tGCI, and then increased back to the basal level at the late phase of reperfusion (Fig. 1D). Similar changes were observed in TIMM23 and HSP60 levels, which instantly decreased at 4 h after reperfusion. However, HPC decreased TOMM20, TIMM23 and HSP60 levels at 50 h after reperfusion. In addition, there was no significant difference in mtTFA level after ischemia with or without hypoxia (Fig. 1D). Next, LC3II/I ratio was determined in both cytoplasmic and mitochondrial fractions to analyze the activation of autophagy in different cellular components of CA1 neurons. Compared with Sham group, LC3II/I ratio increased in both cytoplasmic and mitochondrial fractions of tGCI group. Notably, HPC further increased LC3II/I ratio in mitochondrial fraction at 26 h and 50 h after reperfusion while decreased it in the cytoplasm at 50 h after reperfusion (Fig. 1E, F).

**Fig. 2:**
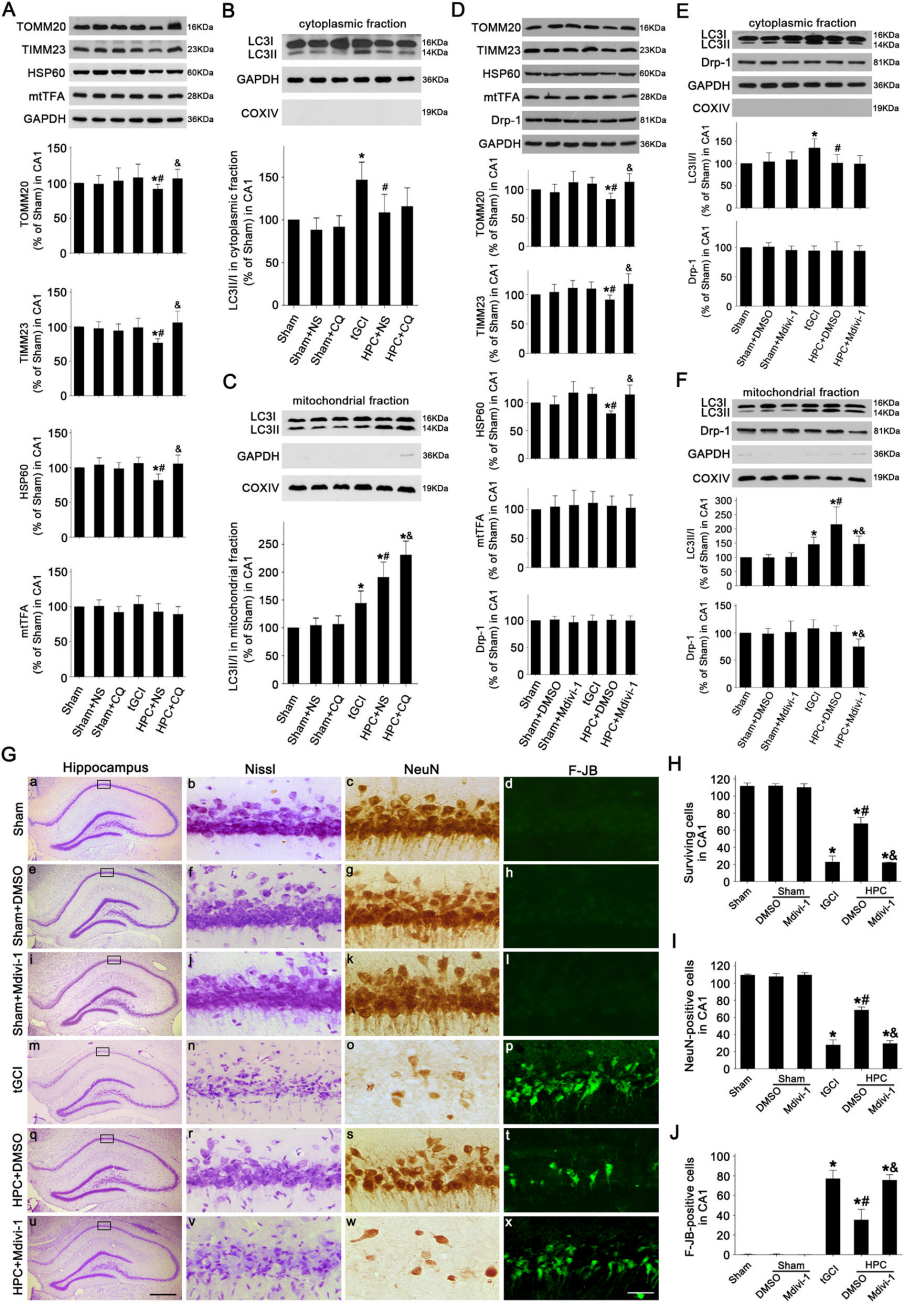
Effects of mitophagy or autophagy inhibition on mitochondrial clearance and neuronal damage in CA1 after ischemia with HPC. **A** Western blot analysis of TOMM20, TIMM23, HSP60 and mtTFA in CA1. The histogram presents the quantitative analyses of TOMM20, TIMM23, HSP60 and mtTFA levels (*n* ≥ 4 in each group). Western blot analysis of LC3 in the cytoplasmic fraction (**B**) and mitochondrial fraction (**C**) of CA1. The histogram presents the quantitative analyses of LC3-II/I ratio. Data are expressed as percentage of value of Sham animals. Each bar represents the mean ± SD. *p < 0.05 vs. Sham group, ^#^*p* < 0.05 vs. tGCI group, and ^&^*p* < 0.05 vs. HPC + NS group. **D** Western blot analysis of TOMM20, TIMM23, HSP60, mtTFA, Drp-1 in CA1. The histogram presents the quantitative analyses of TOMM20, TIMM23, HSP60, mtTFA, Drp-1 levels (*n* ≥ 3 in each group). Western blot analysis of LC3 and Drp-1 in the cytoplasmic fraction (**E**) and mitochondrial fraction (**F**) of CA1. The histogram presents the quantitative analyses of LC3-II/I ratio and the level of Drp-1. Data are expressed as percentage of value of Sham animals. Each bar represents the mean ± SD. *p < 0.05 vs. Sham group, ^#^*p* < 0.05 vs. tGCI group, and ^&^*p* < 0.05 vs. HPC + DMSO group. **G** Representative microphotographs of cresyl violet staining, NeuN immunostaining, and F-JB staining in the hippocampus at 7 days after tGCI with or without Mdivi-1 administration. Sham group (a–d); Sham+DMSO group (e–h), injection with DMSO without ischemia or hypoxia; Sham+Mdivi-1 group (i–l), injection with Mdivi-1 without ischemia or hypoxia; tGCI group (m–p); HPC + DMSO group (q–t), injection with DMSO before tGCI and HPC; HPC + Mdivi-1 group (u–x), injection with Mdivi-1 before tGCI and HPC. Scale bar: a, e, i, m, q, u: 250 μm, b–d, f–h, j–l, n–p, r–t, v–x: 25 μm. **H**–**J** Quantitative analyses of surviving cells, NeuN-positive cells, and F-JB-positive cells in CA1 (*n* = 6 in each group). Each bar represents the mean ± SD. *p < 0.05 vs. Sham group, ^#^*p* < 0.05 vs. tGCI group, and ^&^*p* < 0.05 vs. HPC + DMSO group.

To address whether the effects on TOMM20, TIMM23, HSP60, and mtTFA levels induced by HPC after tGCI are dependent on autophagy-lysosome pathway, lysosome inhibitor chloroquine (CQ) was utilized 2 h before HPC. As shown in Fig. 2A, CQ had no obvious effect on TOMM20, TIMM23, HSP60, mtTFA, and LC3II/I ratio in CA1 of Sham rats. However, compared to the normal saline (NS)-administrated group, CQ reversed the reduction of TOMM20, TIMM23, and HSP60 induced by HPC at 50 h after reperfusion, but had no effect on mtTFA. In addition, CQ had no effect on LC3II/I ratio in the cytoplasmic fraction of Sham rats (Fig. 2B), but it significantly increased LC3II/I ratio in the mitochondrial fraction of HPC rats (Fig. 2C). These observations demonstrated that HPC-mediated the reduction of TOMM20, TIMM23, HSP60 in CA1 after tGCI was attributable to damaged mitochondrial clearance induced by the activation of autophagy-lysosome pathway. Also, the increase of LC3II/I ratio in mitochondrial fraction after CQ administration reconfirmed the activation of autophagy in mitochondrial level after HPC.

Finally, to further test the critical role of mitophagy in HPC-induced neuroprotection against tGCI, Mdivi-1, an inhibitor of mitophagy, was intraventricularly administrated to suppress mitophagy. Because the role of Mdivi-1 in mitophagy suppression depends on its inhibitory regulation on dynamin-related protein 1 (Drp1), Drp1 levels were analyzed after Mdivi-1 administration. As shown in Fig. 2D–F, Mdivi-1 did not alter total and cytoplasmic Drp-1 levels, but significantly reduced mitochondrial Drp-1 in CA1 of HPC rats when compared to vehicle group. Also, Mdivi-1 or vehicle had no impact on the expression of TOMM20, TIMM23, HSP60 and mtTFA, LC3II/I ratio in either cytoplasm or mitochondria, and the number of neurons in CA1 of Sham rats (Fig. 2D–F; G, a–l; H–J). Compared to the vehicle group, Mdivi-1 markedly increased TOMM20, TIMM23, and HSP60 levels, and decreased LC3II/I ratio in mitochondrial fraction after HPC. However, no significant changes were observed regarding mtTFA expression and LC3II/I ratio in the cytoplasm between vehicle and Mdivi-1 group in HPC rats (Fig. 2D–F). In addition, less damaged neurons were found in HPC rats with vehicle adminstration in comparison to tGCI rats (Fig. 2G, m–t; H–J). Moreover, Mdivi-1 led to aggravated neuronal damage at 168 h after reperfusion of HPC rats, accompanied by decreased surviving and neuronal nuclei (NeuN)-positive cells, as well as increased Fluoro-Jade B (F-JB)-positive cells (Fig. 2G, q–x; H–J).

### HPC activates PINK1/Parkin pathway and enhances mitochondrial ubiquitination in hippocampal CA1 after tGCI

**Fig. 3:**
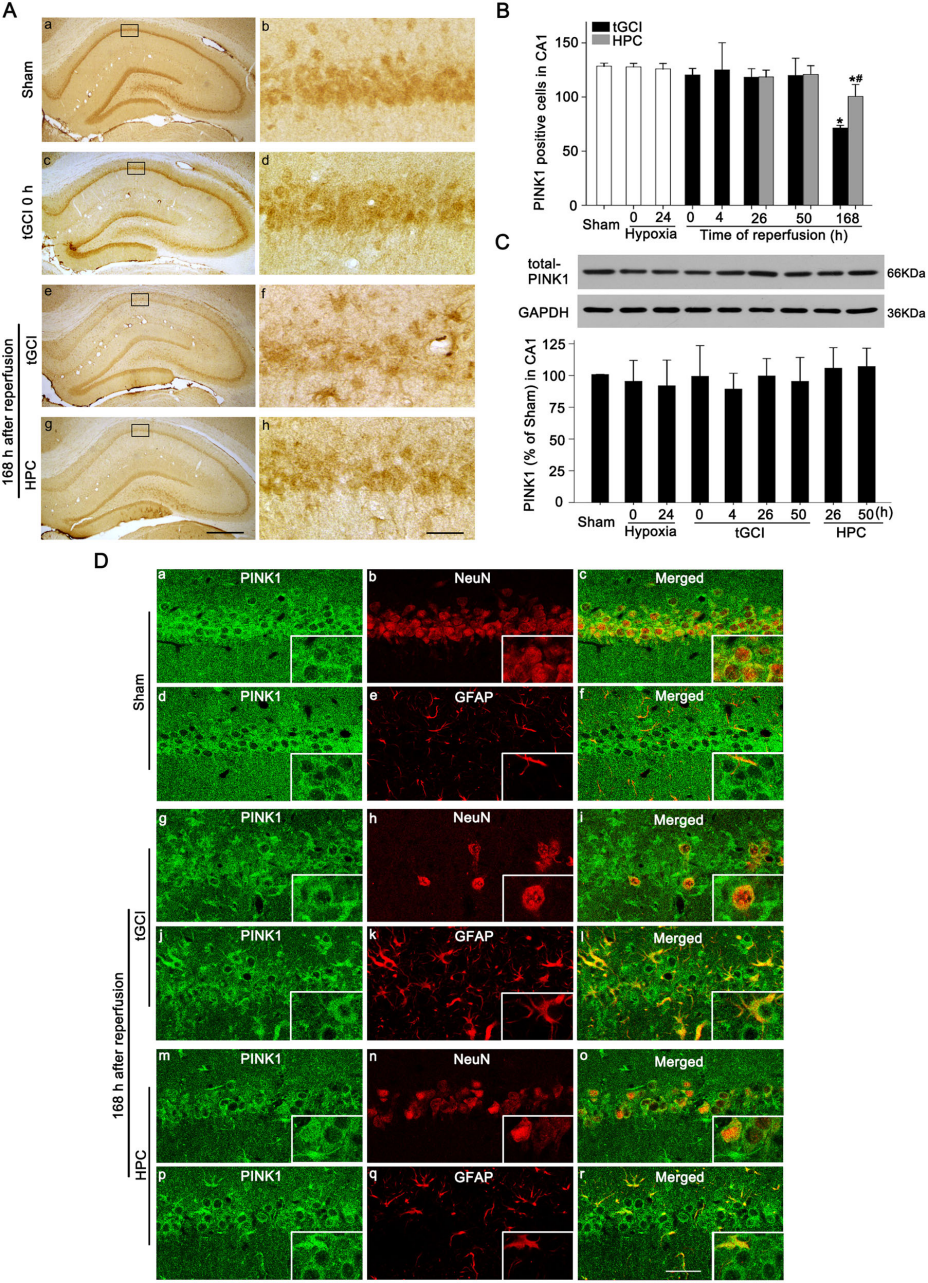
Effect of HPC on the protein expression and cellular localization of PINK1 in CA1 after tGCI. **A** Immunohistochemistry of PINK1 in the rat brains. Representative images show Sham group (a and b), 0 h after reperfusion of tGCI group (c and d), 168 h after reperfusion of tGCI group (e and f) and 168 h after reperfusion of HPC group (g and h), respectively. Scale bar: a, c, e, g: 250 μm; b, d, f, h: 25 μm. **B** Quantitative analyses of PINK1-positive cells in CA1 (*n* = 6 in each group). **C** Western blot analysis of PINK1 in CA1. The histogram presents the quantitative analyses of PINK1 levels (*n* ≥ 4 in each group). Data are expressed as percentage of value of Sham animals. Each bar represents the mean ± SD. *p < 0.05 vs. Sham animals and ^#^*p* < 0.05 vs. tGCI group at the same time point. **D** Representative photomicrographs of fluorescent double staining show that PINK1 (green) mainly distributed in the cytoplasm of NeuN (red)-positive nuclei in CA1 (a–c) but not colocalizated with GFAP (red) (d–f) in Sham group. However, PINK1 (green)-positive cells were mainly GFAP (red)-positive (j–l) rather than NeuN (red) (g–i) in CA1 at 168 h after tGCI. Alternatively, PINK1 (green) located mainly in NeuN (red) (m–o) and partially in GFAP-positive cells (p–r) in CA1 at 168 h after HPC. Scale bar: 50 μm.

**Fig. 4:**
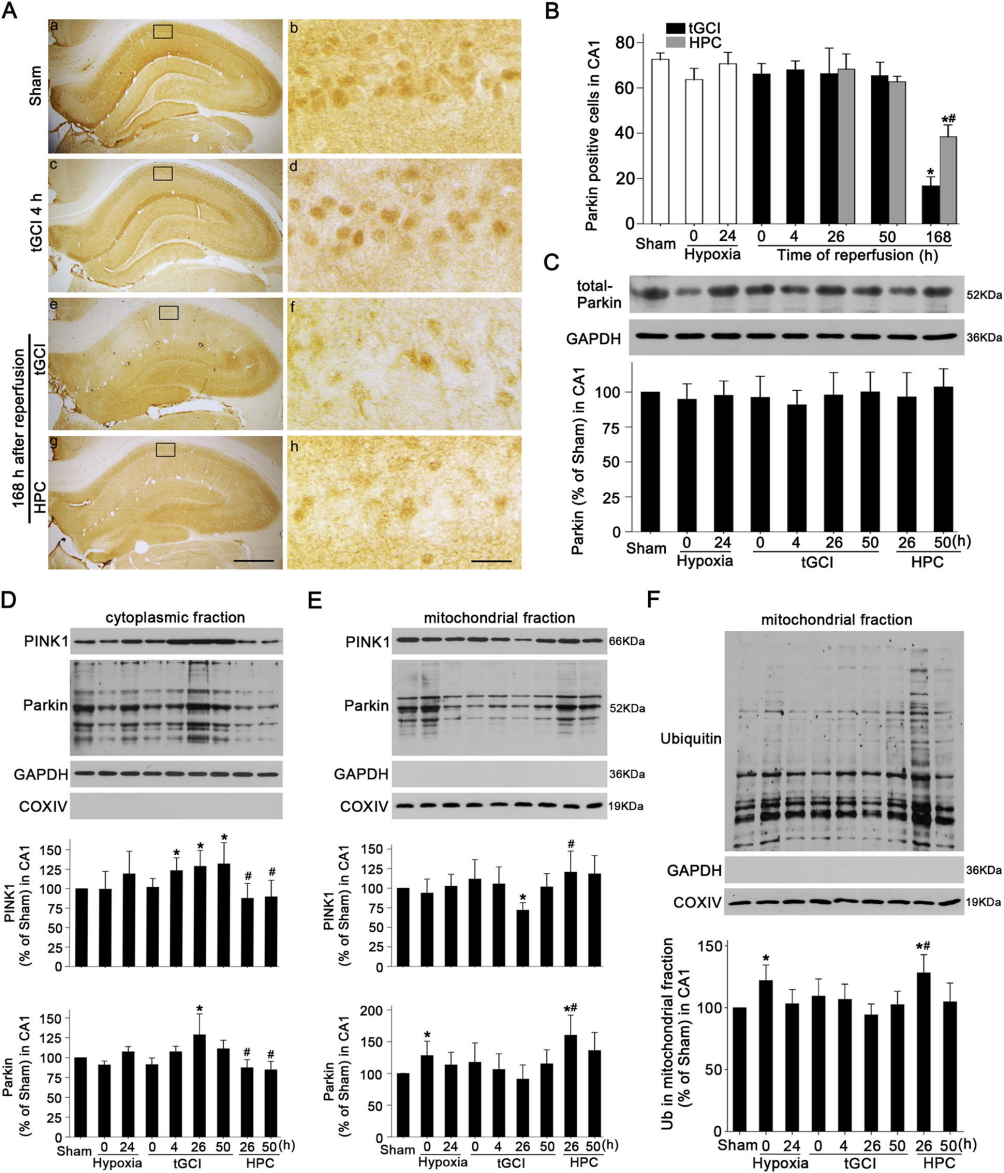
Effects of HPC on the expression of Parkin and the activation of PINK1/Parkin pathway in CA1 after tGCI. **A** Immunohistochemistry of Parkin in the rat brains. Representative images show Sham group (a and b), 4 h after reperfusion of tGCI groups (c and d), 168 h after reperfusion of tGCI groups (e and f) and 168 h after reperfusion of HPC groups (g and h), respectively. Scale bar: a, c, e, g: 250 μm; b, d, f, h: 25 μm. **B** Quantitative analysis of Parkin-positive cells in CA1 (*n* = 6 in each group). **C** Western blot analysis of Parkin in CA1. The histogram presents the quantitative analyses of Parkin levels (*n* = 4 in each group). Western blot analysis of PINK1 and Parkin in the cytoplasmic fraction (**D**) and mitochondrial fraction (**E**) of CA1. The histogram presents the quantitative analyses of PINK1 and Parkin levels (*n* ≥ 4 in each group). **F** Western blot analysis of ubiquitin in the mitochondrial fraction of CA1. The histogram presents the quantitative analyses of ubiquitin levels (*n* ≥ 4 in each group). Data are expressed as percentage of value of Sham animals. Each bar represents the mean ± SD. *p < 0.05 vs. Sham group, ^#^*p* < 0.05 vs. tGCI group at the same time point.

The immunohistochemical assay showed that PINK1-positive cells in Sham rats mainly existed in the pyramidal cell layer with a neuron-like appearance (Fig. 3A, a, b), which was further confirmed by double-fluorescent immunohistochemistry (Fig. 3D, a–f). Most of PINK1-stained cells were NeuN-positive (Fig. 3D, a–c), and only a few were glial fibrillary acidic protein (GFAP)-positive (Fig. 3D, d–f), indicating the predominant expression of PINK1 in CA1 neurons of Sham brains. However, at 168 h after reperfusion, the majority of PINK1-positive cells co-localized with GFAP (Fig. 3D, j–l) which exhibited elongated and irregular nuclei with polymorphic processes (Fig. 3A, e, f) and only a few were NeuN-positive (Fig. 3D, g–i). In contrast, PINK1-positive cells in HPC group were mainly NeuN-positive (Fig. 3D, p–r). There was no difference in PINK1-positive cell number in CA1 between tGCI and HPC groups, except for a decrease at 168 h after reperfusion of tGCI (Fig. 3A, B). Confirmed by western blot, PINK1 level showed no significant differences at 0–50 h after tGCI with or without hypoxia (Fig. 3C). Quantitative analysis of Parkin displayed a similar expression pattern to that of PINK1 (Fig. 4B, C). Parkin-positive cells in Sham or tGCI groups were mainly distributed in the pyramidal cell layer in CA1, with round appearance and homogeneous staining (Fig. 4A, a–d). These cells became heterogeneous with clear processes at 168 h after tGCI (Fig. 4A, e, f).

Further, the subcellular fractions of PINK1 and Parkin were examined. As Shown in Fig. 4D, cyto-PINK1 began to increase progressively at 4 h and peaked at 50 h, whereas cyto-Parkin only increased at 26 h after reperfusion compared to Sham group. Notably, HPC significantly reduced the cytoplasmic accumulation of PINK1 and Parkin after tGCI. Intriguingly, mito-PINK1 significantly decreased at 26 h after reperfusion and then increased to basal level at 50 h. HPC significantly increased mito-PINK1 and mito-Parkin levels at 26 h after tGCI (Fig. 4E). As expected, mitochondrial ubiquitination showed a similar trend, which significantly enhanced at 26 h after reperfusion in HPC group (Fig. 4F).

### The alteration of PINK1 expression regulates Parkin translocation and subsequent mitochondrial clearance in CA1 after tGCI

**Fig. 5:**
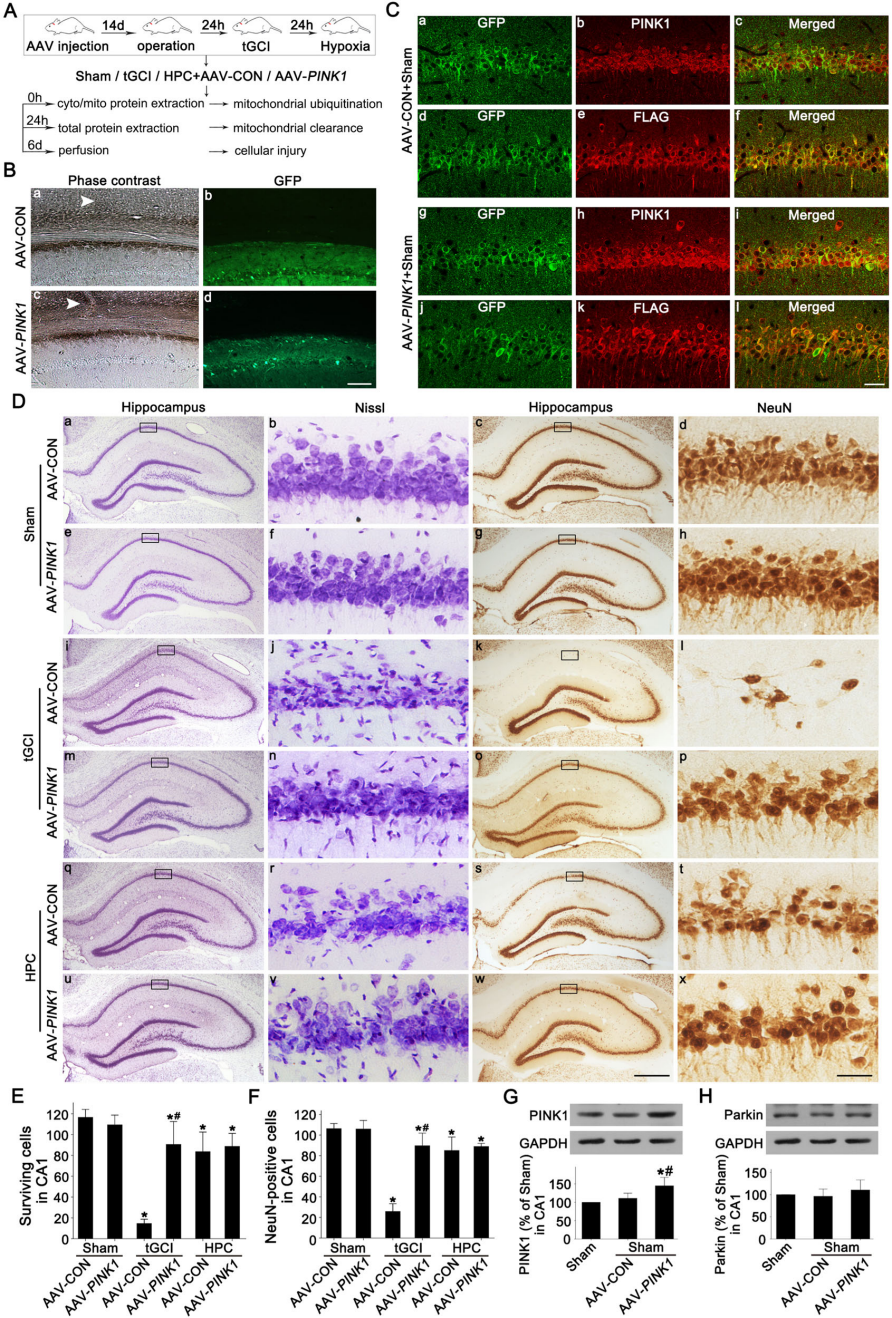
Effects of AAV-PINK1 administration on PINK1 expression and neuronal cell damage in CA1 after ischemia with or without HPC. **A** Study design/timeline of bilateral injection in the dorsal CA1 pyramidal layer with AAV vectors. Mitochondrial ubiquitination, mitochondrial clearance, and cellular injury in CA1 were measured in AAV-injected rats which were subjected to either Sham-operation or tGCI with or without hypoxia. **B** Phase contrast and fluorescent images from coronal sections of CA1 following injection of AAV-CON or AAV-PINK1 in Sham animals. White arrowhead shows the needle trace. Scale bar: 75 μm. **C** Representative photomicrographs show the co-localization of PINK1 (red) (a–c & g–i) or FLAG (red) (d–f & j–l) and GFP (green) in brain sections from Sham animals injected with AAV-CON or AAV-PINK1. GFP: green fluorescence protein; FLAG: flag-tagged protein. Scale bar: 25 μm. **D** Representative microphotographs of cresyl violet staining and NeuN immunostaining in CA1 from rats administered bilaterally with either AAV-CON or AAV-PINK1 at 7 d after reperfusion with or without hypoxia. Boxes indicate that the magnified regions displayed in the right panel. Scale bar: a, c, e, g, i, k, m, o, q, s, u, w: 250 μm; b, d, f, h, j, l, n, p, r, t, v, x: 25 μm. **E**, **F** Quantitative analyses of surviving cells and NeuN-positive cells in CA1. Each bar represents the mean ± SD. *p < 0.05 vs. Sham animals with AAV-CON injection, ^#^*p* < 0.05 vs. tGCI group with AAV-CON injection (*n* = 6 in each group). **G**, **H** Representative immunoblots of PINK1 and Parkin expressions in CA1. The histogram presents the quantitative analyses of PINK1 and Parkin levels. Data are expressed as percentage of value of Sham animals. Each bar represents the mean ± SD. *p < 0.05 vs. Sham animals and ^#^*p* < 0.05 vs. Sham group with AAV-CON injection (*n* ≥ 3 in each group).

**Fig. 6:**
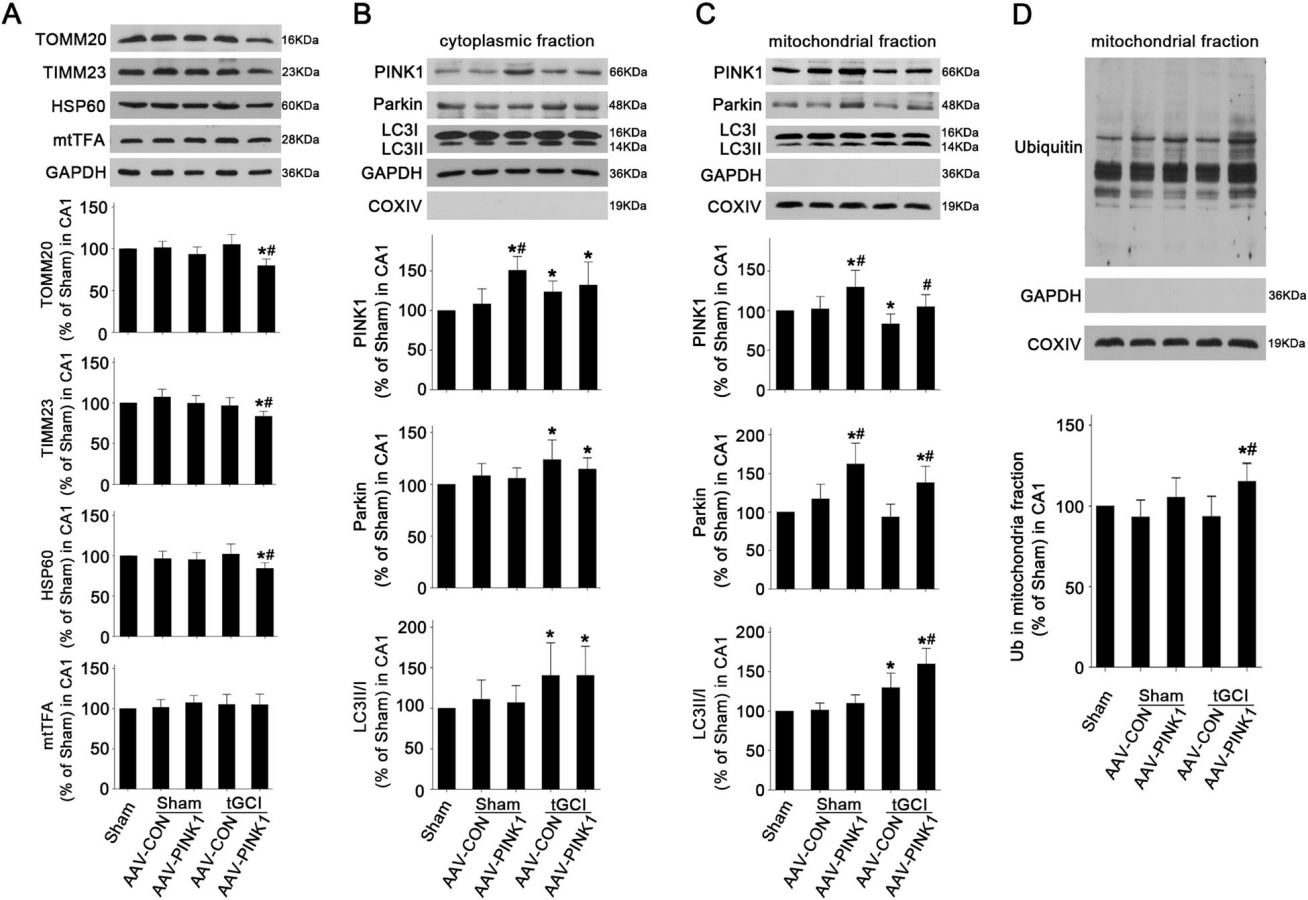
Effects of AAV-PINK1 administration on mitochondrial ubiquitination and mitochondrial clearance in CA1 after ischemia. **A** Western blot analyses of TOMM20, TIMM23, HSP60 and mtTFA in CA1. The histogram presents the quantitative analyses of TOMM20, TIMM23, HSP60, and mtTFA levels. Western blot analyses of PINK1, Parkin, and LC3 in the cytoplasmic fraction (**B**) and mitochondrial fraction (**C**) of CA1. The histogram presents the quantitative analyses of PINK1, Parkin, and the ratio of LC3-II/I. **D** Western blot analysis of ubiquitin in the mitochondrial fraction of CA1. The histogram presents the quantitative analysis of ubiquitin levels. Data are expressed as percentage of value of Sham animals. Each bar represents the mean ± SD. *p < 0.05 vs. Sham group and ^#^*p* < 0.05 vs. the same group with AAV-CON injection (*n* ≥ 4 in each group).

To confirm the role of PINK1 in Parkin translocation to mitochondria and PINK1/Parkin-induced mitophagy in the neuroprotection of HPC, *PINK1*-carried adeno-associated virus (AAV) vector (AAV-PINK1) or scrambled AAV vector (AAV-control, AAV-CON) was administrated into the bilateral CA1 (Fig. 5A). The effectiveness of AAV transfection was verified (Fig. 5B, C). Double-labeled fluorescence showed the colocalization of green fluorescence protein (GFP) and flag-tagged protein (FLAG) after both AAV-CON (Fig. 5C, d–f) and AAV-PINK1 (Fig. 5C, j–l) in Sham rats. In contrast to AAV-CON-administrated rats (Fig. 5C, a–c), GFP-positive cells exhibited stronger PINK1-fluorescent signal after AAV-PINK1 administration (Fig. 5C, g–i). Then, the effects of AAV-PINK1 on the neuronal death after tGCI with or without hypoxia were examined (Fig. 5D–F). AAV-PINK1 or AAV-CON had no impact on the neuronal cell number of Sham rats. Notably, AAV-PINK1 markedly ameliorated neuronal loss compared to AAV-CON after tGCI. However, no additive neuroprotective effect was observed when both AAV-PINK1 and HPC were applied. Besides, PINK1 expression was augmented significantly after AAV-PINK1 administration in Sham rats (Fig. 5G). No obvious change was observed in the Parkin level (Fig. 5H). With AAV-PINK1 administration the reduction of TOMM20, TIMM23, and HSP60 in CA1were promoted at 50 h after reperfusion (Fig. 6A). Compared with AAV-CON group AAV-PINK1 had no any effect on Parkin expression and LC3II/I ratio in cytoplasmic fraction (Fig. 6B). In addition, PINK1 overexpression by AAV-PINK1 increased the level of mito-PINK1, accompanied by increased mito-Parkin and mitochondrial ubiquitination at 26 h of reperfusion after tGCI (Fig. 6C, D).

**Fig. 7:**
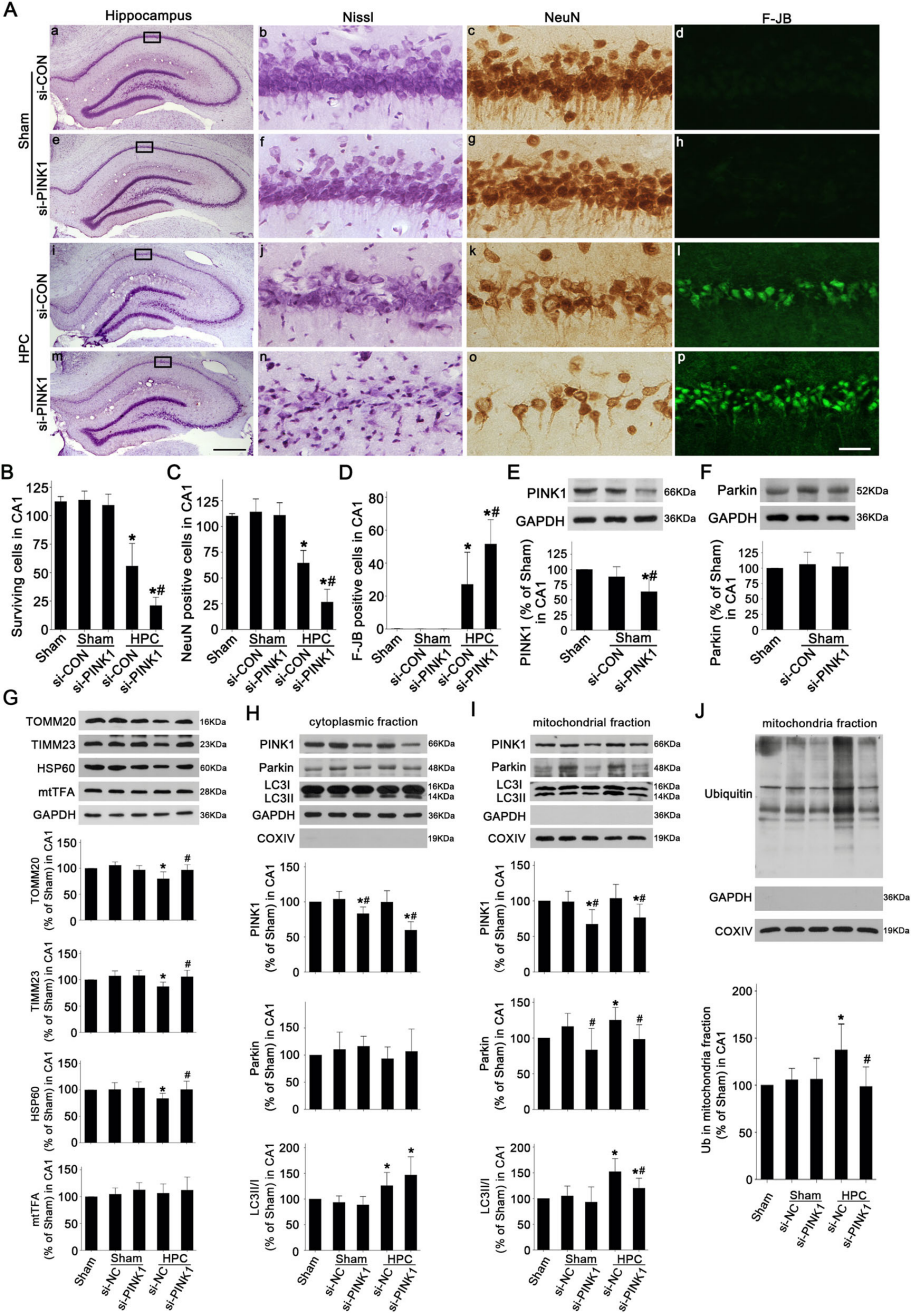
Effects of si-PINK1 administration on neuronal cell damage, mitochondrial ubiquitination, and mitochondrial clearance in CA1 after ischemia with HPC. **A** Representative microphotographs of cresyl violet staining, NeuN immunostaining, and F-JB staining in the hippocampus of HPC rats at 7 days after tGCI with or without si-PINK1 administration. Scale bar: a, e, i, m: 250 μm, b–d, f–h, j–l, n–p: 25 μm. **B**–**D** Quantitative analyses of surviving cells, NeuN-positive cells, and F-JB-positive cells in CA1 (*n* = 6 in each group). Each bar represents the mean ± SD. *p < 0.05 vs. Sham group and ^#^*p* < 0.05 vs. HPC group with si-CON administration. **E**, **F** Effects of si-PINK1 on the expressions of PINK1 and Parkin in CA1 of Sham rats using immunoblot analysis (*n* ≥ 4 in each group). The values are expressed as mean ± SD. *p < 0.05 vs. Sham animals and ^#^*p* < 0.05 vs. Sham group with AAV-CON injection (*n* ≥ 4 in each group). **G** Western blot analyses of TOMM20, TIMM23, HSP60 and mtTFA in CA1. The histogram presents the quantitative analyses of TOMM20, TIMM23, HSP60, and mtTFA levels. Western blot analyses of PINK1, Parkin, and LC3 in the cytoplasmic fraction (**H**) and mitochondrial fraction (**I**) of CA1. The histogram presents the quantitative analyses of PINK1, Parkin, and the ratio of LC3-II/I. **J** Western blot analysis of ubiquitin in the mitochondrial fraction of CA1. The histogram presents the quantitative analysis of ubiquitin levels. Data are expressed as percentage of value of Sham animals. Each bar represents the mean ± SD. *p < 0.05 vs. Sham group and ^#^*p* < 0.05 vs. the same group with si-CON administration (*n* ≥ 4 in each group).

Opposite results were observed after PINK1 siRNA (si-PINK1) administration. Similar to AAV-PINK1, si-PINK1 did not alter the number of surviving, NeuN-positive, and F-JB-positive cells (Fig. 7A, a–h; B–D) in CA1 of Sham rats. However, compared with siRNA-negative control (si-NC), si-PINK1 abolished HPC-induced neuroprotection as evidenced by a dramatic decrease in surviving and NeuN-positive cells after ischemia (Fig. 7A, i–p; B–D). In addition, PINK1 downregulation by si-PINK1 was confirmed by western blot (Fig. 7E) and PINK1 knockdown did not alter Parkin expression of Sham rats (Fig. 7F). Compared to si-NC, si-PINK1 significantly prevented the reduction of TOMM20, TIMM23, and HSP60 in HPC rats, indicating si-PINK1 blocked HPC-induced mitochondrial clearance after tGCI (Fig. 7G). Consistent to AAV-PINK1 injection (Fig. 6A), si-PINK1 had no effect on mtTFA level (Fig. 7G). As shown in Fig. 7H, si-PINK1 reduced cyto-PINK1 level without altering Parkin expression and LC3II/I ratio in the cytoplasmic fraction of HPC rats (Fig. 7H). Oppositely, si-PINK1 led to the reduction of Parkin, LC3II/I ratio, and ubiquitination in mitochondrial fraction of CA1 in HPC group (Fig. 7I, J).

### The alteration of Parkin expression regulates mitochondrial ubiquitination and subsequent mitochondrial clearance in CA1 after tGCI

**Fig. 8:**
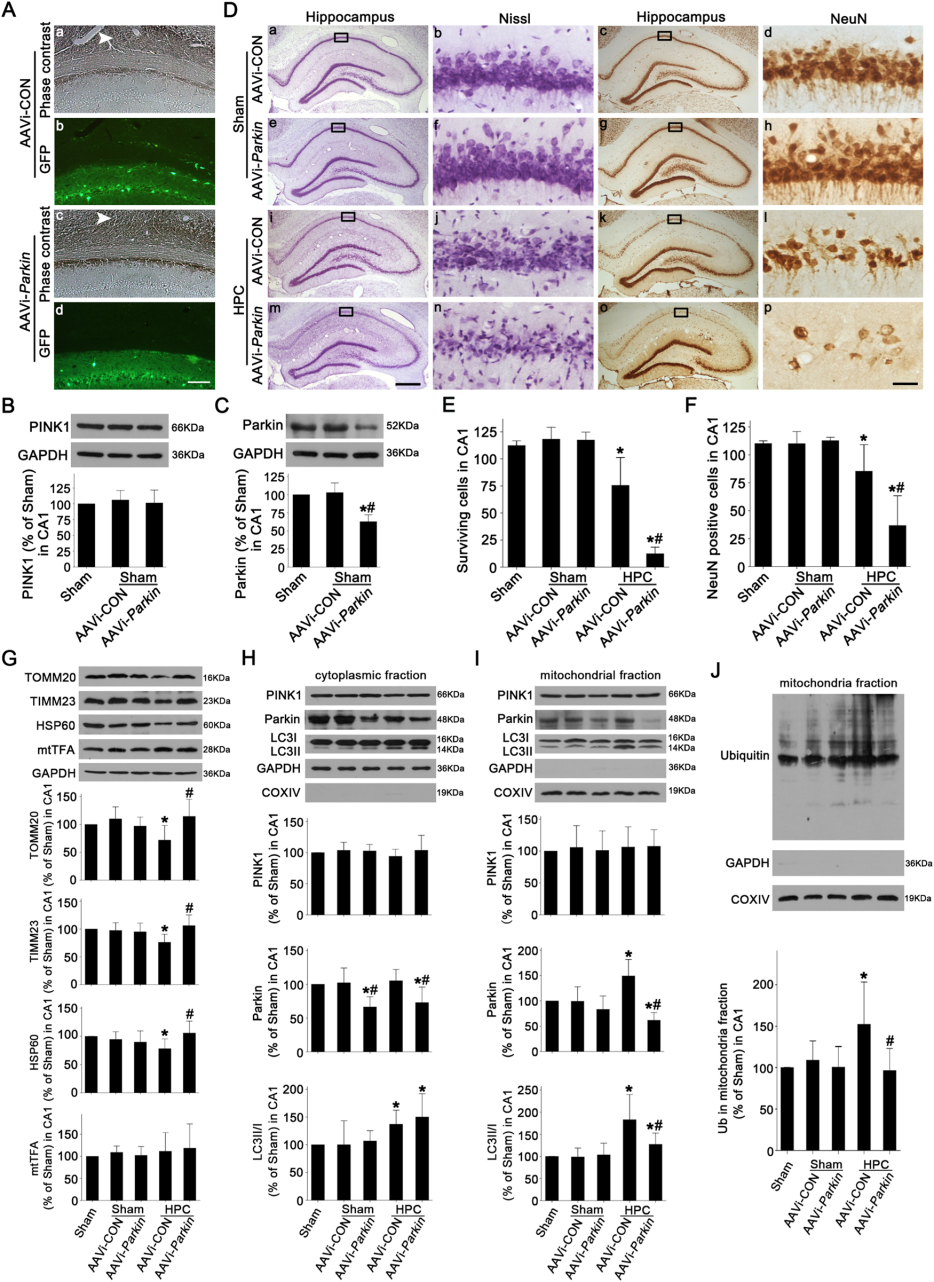
Effects of AAVi-Parkin administration on neuronal cell damage, mitochondrial ubiquitination and mitochondrial clearance in CA1 after ischemia with HPC. **A** Phase contrast and fluorescent images from coronal sections of CA1 following injection of AAVi-CON (a, b) or AAVi-Parkin (c, d) in Sham animals. White arrowhead shows the needle trace. Scale bar: 75 μm. **B**, **C** Representative immunoblots of PINK1 and Parkin expressions in CA1. Data are expressed as percentage of value of Sham animals. Each bar represents the mean ± SD. *p < 0.05 vs. Sham animals and ^#^*p* < 0.05 vs. Sham group with AAVi-CON injection (*n* ≥ 4 in each group). **D** Representative microphotographs of cresyl violet staining and NeuN immunostaining in CA1 from rats administered bilaterally with either AAVi-CON or AAVi-Parkin at 7 d after reperfusion with hypoxia. Boxes indicate that the magnified regions displayed in the right panel. Scale bar: a, c, e, g, i, k, m, o: 250 μm; b, d, f, h, j, l, n, p: 25 μm. **E**, **F** Quantitative analyses of surviving and NeuN-positive cells in CA1. Each bar represents the mean ± SD. *p < 0.05 vs. Sham animals and ^#^*p* < 0.05 vs. the same group with AAVi-CON administration (*n* = 6 in each group). **G** Western blot analyses of TOMM20, TIMM23, HSP60 and mtTFA in CA1. The histogram presents the quantitative analyses of TOMM20, TIMM23, HSP60, and mtTFA levels. Western blot analyses of PINK1, Parkin, and LC3 in the cytoplasmic fraction (**H**) and mitochondrial fraction (**I**) of CA1. The histogram presents the quantitative analyses of PINK1, Parkin, and the ratio of LC3-II/I. **J** Western blot analysis of ubiquitin in the mitochondrial fraction of CA1. The histogram presents the quantitative analysis of ubiquitin levels. Data are expressed as percentage of value of Sham animals. Each bar represents the mean ± SD. *p < 0.05 vs. Sham group and ^#^*p* < 0.05 vs. the same group with AAVi-CON injection (*n* ≥ 4 in each group).

To test the role of Parkin in mitochondrial ubiquitination in HPC-induced neuroprotection, AAV-mediated *Prkn* small-interfering RNA (AAVi-Parkin) or AAV vector carrying scrambled RNAi (AAVi-CON) was injected in CA1. Fluorescent images confirmed the effective transfection of AAV in CA1 (Fig. 8A). AAVi-Parkin significantly downregulated Parkin expression without altering PINK1 of Sham rats (Fig. 8B, C). Consistent with si-PINK1, AAVi-Parkin or AAVi-CON did not alter surviving and NeuN-positive cell number of Sham rats (Fig. 8D, a–h; E, F), whereas AAVi-Parkin dramatically aggravated neuronal damage in CA1 after HPC (Fig. 8D, i–p; E, F). Furthermore, HPC-induced reduction of TOMM20, TIMM23, and HSP60 was abrogated by AAVi-Parkin (Fig. 8G). Similarly, Parkin downregulation had no effects on mtTFA level (Fig. 8G), cyto-PINK1 and mito-PINK1, and LC3II/I ratio in the cytoplasmic fraction of CA1 (Fig. 8H, I). However, compared to AAVi-CON group, AAVi-Parkin significantly suppressed the increase of LC3II/I ratio and ubiquitination level in the mitochondrial fraction of HPC group (Fig. 8I, J).

## Discussion

The current study provides the first evidence for the critical role of mitophagy in the protective effect of HPC in hippocampus CA1 against tGCI. As demonstrated, HPC-promoted mitochondrial clearance via mitophagy at the late phase of reperfusion after tGCI. Inhibiting mitophagy by inhibitors (Mdivi-1 and CQ) eliminated the reduction of TOMM20, TIMM23, and HSP60, and the neuroprotection of HPC. Mechanistically, HPC–promoted mitophagy was dependent on the activation of PINK1/Parkin pathway. PINK1 overexpression in CA1 enhanced Parkin translocation to mitochondria, thereby promoting mitochondrial ubiquitination. On the contrary, the downregulation of PINK1 and Parkin impeded HPC-induced activation of PINK1/Parkin pathway, inhibited mitochondrial clearance, and consequently aggravated neuronal damage in CA1 after tGCI.

Mitochondrial dysfunction is involved in multiple pathophysiological processes after cerebral ischemia [3], including oxidative stress, endoplasmic reticulum stress, and subsequent apoptosis. Previously we have reported a remarkable number of dilated endoplasmic reticulum and swollen mitochondria [6], a progressive increase of reactive oxygen species [23], and the activation of caspase-3 and caspase-9 in CA1 neurons after tGCI [24, 25]. As an endogenous adaptive response, mitophagy is reported to affect neuronal fates in the ischemic brain through selective mitochondrial turnover [9, 12, 26, 27]. In this study, we demonstrated that at the early phase of reperfusion, mitochondrial mass was reduced in CA1, as revealed by a decrease of TOMM20 at 0–4 h and the reduction of TIMM23 and HSP60 at 4 h of reperfusion. As reported, the degradation of specific OMM proteins can inhibit the fusion of damaged mitochondria with healthy mitochondria, thereby segregating impaired mitochondria from the healthy mitochondria network to facilitate their selective elimination [28]. Moreover, rapid degradation of OMM proteins is a prerequisite for mitophagy [29]. These might be the reasons why the degradation of TOMM20 was prior to that of TIMM23 and HSP60 at the early phase of reperfusion after tGCI in our study. However, the decline of mitochondrial mass was disrupted at the late phase of reperfusion, whereas HPC facilitated mitochondrial clearance after tGCI. The engulfment of mitochondria by vacuolar structures and the increase of LC3II/I ratio in the mitochondrial fraction, two main features of mitophagy activation and mitophagosomes formation [8, 30], were verified to be enhanced in CA1 of HPC groups at the late phase of reperfusion after tGCI. Generally, as a key regulator of mtDNA copy number in mammals, mtTFA regulates mtDNA replication and transcription [31]. Therefore, mtTFA is important in representing the activation of mitochondrial biogenesis. Considering that no significant difference in mtTFA level was observed between tGCI and HPC groups, HPC-induced decline of mitochondrial mass was possibly ascribed to the increase of mitochondrial turnover rather than the suppression of mitochondrial biogenesis. To further confirm this hypothesis, Mdivi-1 or CQ was administrated before HPC. The results showed that Mdivi-1 or CQ administration prevented the reduction of TOMM20, TIMM23, and HSP60 in CA1 and abrogated HPC-induced neuroprotection. These findings suggested that HPC protected neurons via promoting mitophagy at the late phase of reperfusion after tGCI.

In addition to mitophagy, Drp1, a crucial mediator of mitochondrial fission, mediates neuronal death after transient focal cerebral ischemia [32]. Our previous study showed that tGCI reduced phosphorylated Drp1 at serine 637 in CA1 [33]. Intriguingly, Drp1 dephosphorylation at serine 637 enhances mitochondrial fission [34–36], which helps autophagosomes to engulf mitochondria [37]. Hence, the transient decrease of mitochondrial mass at the early phase of reperfusion might result from mitophagy promoted by Drp1 dephosphorylation at serine 637. However, the elimination of damaged mitochondria after tGCI was blocked at the late phase of reperfusion [33], revealing that there might be other mechanisms responsible for mitophagy regulation at this phase.

Actually, PINK1/Parkin pathway plays a key role in promoting the clearance of impaired mitochondria [38]. PINK1 and Parkin are two proteins stably expressed in the nervous system of human being [39, 40], and crucial for neuronal survival functioning as multipurpose proteins in a variety of toxic insults [41, 42]. Notably, PINK1/Parkin pathway has been arisen extensive attention in exploring the interplay between hypoxia-related mitophagy and cerebral ischemia [8, 19, 22]. As a sensor of mitochondrial damage, PINK1 accumulates in the OMM and recruits cytosolic Parkin to damaged mitochondria. Then Parkin stimulates the degradation of mitochondria by ubiquitination of mitochondrial proteins [43]. Cerebral ischemia would alter PINK1 and Parkin expression [19, 22]. Demyanenko et al. demonstrated that expression of Parkin and PINK1 were increased in the cortical penumbra at 1 h after photothrombotic infarction in Wistar rats [44]. In contrast, Mengesdorf et al. reported that transient focal ischemia markedly decreased Parkin protein expression within the first 24 h of reperfusion in adult mice after 1 h of MCAO [45]. The discrepancies in the alterations of Parkin and PINK1 levels after ischemic stimuli may due to the differences in the severity of ischemic stimuli, the selection of the experimental model, the duration of reperfusion, and the region of interest for the study [19–21, 46, 47]. In the current study, no significant differences in total PINK1 and Parkin expressions were detected in CA1 after tGCI. However, HPC-promoted PINK1 and Parkin translocation to mitochondria, accompanied by the increased mitochondrial ubiquitination and subsequent mitochondrial clearance after tGCI. Moreover, PINK1/Parkin-induced mitophagy is protective for the hippocampus in vivo and in vitro against cerebral ischemia [20, 21], which mainly arises from the suppression of oxidative stress, alleviation of neuronal apoptosis [48], and improvement of mitochondrial function [22]. Consistently, we found that PINK1 silencing abrogated the activation of PINK1/Parkin pathway, inhibited the degradation of damaged mitochondria, and finally eliminated the neuroprotection induced by HPC after tGCI. In addition, PINK1 overexpression mimicked the role of HPC in mitophagy and offered neuroprotection against tGCI.

It was documented that Parkin translocation mediates mitochondrial ubiquitination as a mechanism for mitochondrial priming, a critical step that “preparing” the mitochondria for autophagic recognition to promote autophagic removal of mitochondria [49]. In our study, HPC-promoted mitochondrial ubiquitination in CA1 after tGCI and subsequent damaged mitochondria clearance. PINK1 knockdown with siRNA, however, eliminated HPC-induced mitochondrial clearance after tGCI. These findings indicated the involvement of PINK1/Parkin pathway in mitochondrial clearance. After being activated by PINK1, Parkin modifies many cytosolic and OMM proteins with K48- and K63-linked ubiquitin chains [29]. In addition, Parkin is capable to form non-canonical K6- and K11-linked chains [16]. Interestingly, Parkin-generated polyubiquitin chains on mitochondria likely lead to degradation of OMM proteins by proteasome [29, 50]. Moreover, the removal of OMM proteins via Parkin-mediated activation of ubiquitin-proteasome system appears to be critical for mitophagy [28, 29]. Besides, Parkin-dependent poly-ubiquitination contributes to the recruitment of ubiquitin-binding autophagy receptor, such as p62/SQSTM1 which is known to connect the ubiquitin system and the autophagic machinery [51]. These receptors ensure selective autophagy by sequestering specific ubiquitinated components into an autophagosome before delivering to lysosome for degeneration [52]. In short, for the first time our results supported that HPC-induced mitochondrial ubiquitination by the activation of PINK1/Parkin pathway led to subsequent mitochondrial clearance in CA1 after tGCI.

In summary, HPC activated PINK1/Parkin pathway to promote mitochondrial ubiquitination and subsequent mitochondrial clearance in CA1 after tGCI. Despite that much more endeavor is needed to further elucidate the regulatory mechanisms of mitophagy involved in this model, to a certain extent, our study has provided new insights into the involvement of PINK1/Parkin-dependent mitophagy in the neuroprotection induced by HPC against tGCI. Therefore, these findings would bring forth the possibility that the targeted manipulation of mitophagy may be of clinical significance for the novel therapeutic interventions to cerebral ischemia.

## Materials and methods

### Animals

Adult male Wistar rats weighing 220–280 g (purchased from Southern Medical University, Guangzhou, China) were utilized in this study. All animal-related experiments were operated on the basis of the *Animal Research: Reporting* in vivo *Experiments (ARRIVE)* guidelines and were supervised by the Animals Care and Use Committee of Guangzhou Medical University (Guangzhou, China). All animals were housed in a temperature-controlled (21–23 °C) and 12-h light/dark cycle environment with ad libitum water and food. Every effort was made to minimize the number and the suffering of animals. Animals went through randomization using a random number table and were allocated to different experimental groups according to the standard procedures.

In this study, 702 rats were used. Six rats in each group from Sham, 26 h and 50 h after tGCI with and without HPC were perfused for TEM analysis. Six rats for total protein detection and 10 rats for cytosolic/mitochondrial proteins were sacrificed in each group from Sham, at 0, 4, 26, and 50 h after tGCI with and without HPC. Five rats in each group were used for total protein detection at 50 h after reperfusion and six rats were sacrificed for cytosolic/mitochondrial proteins detection at 26 h after reperfusion with CQ, Mdivi-1, AAV-PINK1, and si-PINK1 injection respectively. Another four rats in each group were used for total Drp-1 detection at 50 h after reperfusion and five rats were sacrificed for cytosolic/mitochondrial Drp-1 detection at 26 h after reperfusion with Mdivi-1 injection. For Mdivi-1 and AAV-PINK1 injection, six rats were perfused in Sham, tGCI, and HPC groups at 168 h after reperfusion. Six rats were perfused to measure cellular injury after si-PINK1 and AAVi-Parkin injection in Sham and HPC groups at 168 h after reperfusion. Five rats in each group were used for total protein detection at 50 h after reperfusion and seven rats were sacrificed for cytosolic/mitochondrial proteins detection at 26 h after reperfusion with AAVi-Parkin injection.

Besides, six rats died during the surgery. Five rats in the tGCI and four in the HPC groups died during the tGCI procedure. Four rats in the tGCI and three in the HPC groups died after tGCI. Two rats died after hypoxia. Four rats died after intracerebroventricular injection, one died after intraperitoneal administration, two died after AAV injection, two died after siRNA administration, and three died during AAVi injection.

### Experimental models

A four-vessel occlusion method was used to produce tGCI model [53]. Briefly, anesthesia for rats was induced with 3–4% isoflurane in a chamber and maintained with 2–3% isoflurane in pure oxygen (800 ml/min) with a mask during the surgery. Bilateral vertebral arteries were blocked by electrocauterization and bilateral common carotid arteries were isolated. Then a teflon/silastic occluding device was assembled loosely around each carotid artery without blood flow interruption. Global cerebral ischemia was induced by occluding both common carotid arteries for ten minutes after 24 h of surgery in awake rats. Rats that had mydriasis and lost righting reflex within 1 min were enrolled for the following experiments. Rectal temperature was maintained at 37–38 °C throughout the whole process by using heating pad. Sham rats received the same surgical operation without 10-min occlusion of common carotid arteries. Rats that convulsed during ischemia or post-ischemia were excluded from this study.

Rats were treated with the hypoxic postconditioning process at 24 h after tGCI [23, 54]. Concisely, animals were placed in a sealed chamber of 9000 cm^3^ with continuously flowing mixed-gas (8% O_2_ + 92% N_2_) for 120 min at room temperature of 23–25 °C. For sham-operated, hypoxia-treated groups, animals were exposed to 120-min hypoxia at 24 h after Sham-operation without tGCI.

### Transmission electron microscopy

For electronic microscopic analysis, brain samples were processed as previously described [6]. Briefly, samples were pre-fixed in 4% paraformaldehyde (pH 7.4) containing 2.5% glutaraldehyde and post-fixed in 0.1 M sodium cacodylate-buffered 1% O_s_O_4_ solution. After dehydrated with a step-wise ethanol gradient solution, samples were incubated with propylene oxide, then impregnated with a mixture of propylene oxide/Spurr (1:1) and embedded in Spurr resin. Ultrathin sections were examined via transmission electron microscope (TEM, Tecnai G2 Spirit, FEI). Only the vesicles that are appropriate in size, retain a double-membrane structure, and contain integral or remnants of mitochondrial cristae were counted in our study. Four rats were used at each group and four tissue samples were taken from thin sections of each animal. Ten electron micrographs of CA1 pyramidal neurons were taken per section with ×6800 magnification. Morphometrical measurements of mitophagosomes were carried out using the point-counting method. Data from each electron micrograph was used to calculate the mean and the variation. The cytoplasmic volume fractions of the mitophagic vacuole were expressed as percentage of the cytoplasmic volume. Data were quantified bilaterally in sections from each brain and assessed blindly.

### Western blot

Animals were sacrificed at 0, 4, 26, and 50 h after reperfusion of tGCI with or without HPC (*n* ≥ 5 in each group), respectively. After incising brain samples into 2-mm coronal slices, bilateral hippocampal CA1 subregions were quickly divided under the stereomicroscope. Total protein extraction of CA1 subregion was performed as previously described [54]. The cytosolic and mitochondrial proteins of CA1 were extracted with Mitochondria/Cytosol Fractionation Kit (Cat# ab65320, Abcam) according to the manufacturer’s protocol. The purity of each fraction was measured by the expression of corresponding reporters, including cytochrome c oxidase IV (COXIV) and glyceraldehyde 3-phosphate dehydrogenase (GADPH). Western blot was conducted as described previously [54] with the primary antibodies including TOMM20 (1:500; Cat# ab56783, Abcam), TIMM23 (1:3000; Cat# 611223, BD Biosciences), HSP60 (1:6000; Cat# 4870, Cell Signaling Technology), mtTFA (1:3000; Cat# ab131607, Abcam), LC3 (1:10000; Cat# 4870, Novus Biologicals), PINK1 (1:1000; Cat# ab23707, Abcam), Parkin (1:1000; Cat# ab23707, Abcam), ubiquitin (Ub) (1:1000; Cat# ab7780, Abcam), GADPH (1:10,000; Cat# 60004-I-Ig, Proteintech Group), COXIV (1:20,000; Novus Biologicals). Densitometric analysis for the quantification of the bands was carried out with ImageJ (NIH, Bethesda, MD, United States). Relative optical densities of protein bands were calibrated with GAPDH or COXIV and normalized to those in Sham rats.

### Administration of drugs

Intracerebroventricular injection via the cannula affixed to the right parietal skull (1.0 mm posterior to bregma, 1.5 mm lateral to bregma, and 3.6 mm below the dura) was performed as previously described [54], at a speed of 60 μl/h. In short, Mdivi-1 (1 mg/kg, 3 μl; Cat# M0199, MilliporeSigma) or vehicle (isometric 25% dimethyl sulfoxide, DMSO) was administrated at 0 h after reperfusion of tGCI and 30 min before HPC. CQ (60 mg/kg, 10 mg/ml, Sigma) or NS was administered intraperitoneally at 2 h before HPC.

### Assessment of cellular injury

At 168 h after reperfusion of tGCI, rats were perfused intracardially with NS and 4% cold paraformaldehyde in PBS. After post-fixation with 10, 20, and 30% sucrose in the same fixative, brain samples were frozen at −22 °C and sliced into coronal 30 μm-thick sections with cryotome (CI950, Leica, Wetzlar, Hessen, Germany). Then sections of the dorsal hippocampus (between anterior-posterior (AP) 4.8 and 5.8 mm, interaural or AP 3.3–3.4 mm, bregma) were used to determine cellular damage in CA1 subregion.

Nissl, NeuN, and F-JB staining were performed following the protocol described previously [54]. Nissl-stained cells and NeuN-were observed under a light microscope with ×660 magnification. F-JB stained images were captured with a fluorescent microscope (DMI 4000B, Leica Microsystems, Wetzlar, Hessen, Germany). Cells in CA1 were analyzed within four non-repeated random fields of 0.037 mm². Data were quantified bilaterally in four sections from each animal and were assessed in a blind procedure.

### Immunohistochemistry

The avidin-biotin complex peroxidase method was used to perform single-label immunohistochemistry [54]. In brief, brain slices were prepared as abovementioned from rats sacrificed at 0, 4, 26, 50, and 168 h after reperfusion of tGCI with or without HPC (*n* ≥ 6 in each group). The sections used to assess cellular injury were also utilized to conduct immunohistochemistry. These sections of the dorsal hippocampus were treated with 3% hydrogen peroxide for 30 min, 5% normal serum for 1 h, and then incubated overnight at 4 °C with primary antibodies including NeuN (1:6000; Cat# MAB377; MilliporeSigma), PINK1 (1:200) and Parkin (1:3500). The number of immunopositive cells was assessed blindly by the total number of four non-repeated random fields (0.037 mm^2^/field × 4 = 0.148 mm^2^ in total) in CA1. In addition, 4 sections /rat were evaluated.

Double-fluorescent immunohistochemistry was carried out to confirm the expression pattern of PINK1 in different groups. NeuN and GFAP were used to identify neuronal nuclei and astrocytes, respectively. Antibodies used in these studies include PINK1 (1:100), NeuN (1:1000), GFAP (1:4000; Cat# MAB360; MilliporeSigma), Cy3-conjugated goat anti-mouse IgG antibody (1:100; Cat# AP124C; MilliporeSigma), and goat Anti-Rabbit IgG H&L (Alexa Fluor® 488) (1:100; Cat# ab150077, Abcam). Fluorescent images were obtained with a confocal laser microscope (SP8, Leica Microsystems).

For negative control, brain slices went through the same experimental procedure of single-label immunohistochemistry or double-fluorescent immunohistochemistry without adding corresponding primary antibodies.

### PINK1 RNAi knockdown

To inhibit PINK1 expression, pre-designed PINK1-siRNA from MISSION^®^ siRNA (Sigma-Aldrich Ltd, St. Louis, MO, USA) was bilaterally injected into hippocampal CA1 region (3.5 mm posterior to bregma, 2.3 mm lateral to bregma, 2.5 mm below the dura). The sequences of si-PINK1 were as follows: sense, 5′GCUGCAAUGCCGCUGUGUA3′, antisense, 5′ UACACGCGGCAUUGCAGC3′. For si-NC, the sequences were as follows: sense, 5′GAUCAUACGUGCGAUCAG A3′, antisense, 5′UCUGAUCGCACGUAUGAU C3′ [48]. Hemagglutinating virus of Japan HVJ-E vector kit (CosmoBio, Tokyo, Japan) was used to deliver siRNA into CA1 region [6]. According to the manufacturer’s protocols, HVJ-E was incorporated with si-PINK1 to yield an HVJ-E si-PINK1 complex (vector). The vectors were introduced into CA1 region by the membrane-fusion activity of fusion protein. PINK1 siRNAs or negative control siRNA (10 μl; 0.5 OD) was combined with HVJ-E, respectively. The complexes in PBS were administered by stereotaxic injection at 24 h before tGCI. A 5-μl volume containing 0.25 OD siRNA was injected into the CA1 region using a 10-μl Hamilton syringe at a flow rate of 0.3 μl/min. For immunoblot analysis, rats were sacrificed at 26 and 50 h after reperfusion of tGCI in HPC group or 50 and 74 h after injection of vectors in the sham-operated group.

### Adeno-associated virus construction and administration

Plasmids containing the sequence of rat *PINK1* (GenBank accession number NM_001106694), ATGGCGGTGCGACAGGCACTGGGCCGAGGCCTGCAGCTG GGTCGGGCGCTGCTGCTGCGCTTCGCTCCCAAGCCGGGCCCGGTGTCAGGCTGGGGCAAGCCCGGCCCCGGTGCGGCCTGGGGCCGCGGAGAGCGTCCCGGCCGGGTCTCAAGCCCGGGAGCACAGCCGCGTCCGCTCGGGCTCCCCCTCCCGGACCGCTACCGCTTCTTCCGCCAGTCGGTGGCTGGGCTGGCGGCGCGAATCCAGCGGCAGTTCGTGGTGCGGGCCCGAGGCGGCGCAGGGCCTTGCGGCCGAGCAGTCTTCCTGGCCTTCGGACTGGGGTTGGGGCTGATCGAGGAGAAGCAGGCGGAGAGCCGGAGGGCCGCCTCGGCCTGTCAGGAGATCCAGGCAATTTTTACACAGAAAAACAAGCAAGTGTCTGACCCACTGGACACACGACGTTGGCAGGGCTTCCGCCTGGAGGATTATCTGATAGGACAGGCCATCGGCAAGGGCTGCAATGCCGCTGTGTATGAAGCCACCATGCCCACACTGCCCCAGCACCTGGAGAAGGCCAAACACCTTGGCCTTCTAGGAAAAGGCCCAGATGTCGTCTCAAAGGGAGCAGATGGGGAGCAGGCTCCAGGGGCCCCCGCCTTCCCCTTTGCCATCAAAATGATGTGGAATATCTCGGCAGGATCCTCCAGCGAAGCCATCTTAAGCAAAATGAGCCAGGAGCTGGAAGCCTTGGGTTCAGCAAACAGGAAGGGCACCCTTCAACAGTTCAGGCGG, and a negative control vector (CON323) were designed by Genechem (Shanghai, China). Briefly, *PINK1* sequence was inserted into *Eco*RI and *Bam*HI sites of the hSyn promoter-MCS-EGFP-3FLAG-SV40 PolyA (GV466) AAV vector. The titers were ~1.09 × 10^13^ TU/ml. A 2-μl volume containing 1.09 × 10^10^ TU of particles was bilaterally injected into hippocampal CA1 region.

Three small-interfering RNA sequences targeted rat *Parkin* (*Prkn*, GenBank accession number NM_031970) and a negative control vector (CON323) were designed by Genechem (Shanghai, China). The sequences for *Prkn*-RNAi 1, 2, 3 were ACCGCTAGCTAACTGGAGGCTTGCTGAAGGCTGTATGCTGAACGATACTCTGTTGTTCCAGGTTTTGG; ACCAAGCTTGGGCCATTTGTTCCATGTGAGTGCT AGTAACAGGCCTTGTGTCCTGAACGATA and TCTGTTGTTCCAGGTTTTGGC CACTGACTGACCTGGAACAAGAGTATCGTTCAGGACACAAGGC, respectively. Briefly, RNAi was inserted into *Eco*RI and *Bam*HI sites of the hSyn promoter-EGFP-MIR155(MCS)-SV40 PolyA (GV680) AAV8 vector according to the manufacture’s instruction. The shuttle vector and viral packaging system were cotransfected into AAV-293 cells, which derived from HEK293 cells, to produce recombinant AAV particles. The best-performing *Prkn*-RNAi sequence was ACCGCTAGCTAACTGGAGGCTTGCTGAAGGCTGTATGCTGAACGATACTCTGTTGTTCCAGGTTTTGG (*Prkn*-RNAi 1) with maximal inhibition. Therefore, *Prkn*-RNAi 1 was used for subsequent experiments. The titers were ~1.55 × 10^12^ TU/mL. A 5-μl volume containing 7.75 × 10^9^ TU of particles was bilaterally injected into CA1 region.

Rats were allowed to recover for up to 14 days after AAV injection to ensure sufficient gene expression before being used for subsequent experiments.

### Statistical analysis

Data analysis was performed with Statistical Package for Social Sciences for Windows, version 17.0 (SPSS, Inc, Chicago, Illinois, USA). All variables were expressed as mean ± SD. Two-way analysis of variance (ANOVA) was utilized to compare the numbers of immunopositive cells or the optical densities of protein bands between HPC and tGCI rats. One way-ANOVA was used to compare the number of immunopositive cells or the optical densities of protein bands between tGCI and Sham rats. Analyses were followed by a Bonferroni or Tamhane’s T2 post hoc test. Differences were considered statistically significant when *p* < 0.05.
